# Specific intervention program for ARFID comorbid with ASD in a Children’s Youth Autism Day Hospital

**DOI:** 10.1192/j.eurpsy.2024.922

**Published:** 2024-08-27

**Authors:** A. Alvarez, N. Santamaria, V. Bote, R. Medina, B. Sanchez, I. Mendez, J. A. Monreal, A. Hervas

**Affiliations:** ^1^University Hospital Mutua Terrassa, Terrassa, Spain; ^2^Mental Health, University Hospital Mutua Terrassa, Terrassa, Spain

## Abstract

**Introduction:**

Children and adolescents with ASD are more likely to have eating problems compared to the general population of the same age, one of the disorders whose prevalence is increased in people with ASD is avoidant/restrictive eating disorder Food (ARFID) ARFID is characterized by a lack of interest in eating or avoidance of food intake, which in the case of people with ASD is usually related to impaired sensory processing and cognitive rigidity. For this reason, the Autism Day Hospital carries out a specific food intervention program.

**Objectives:**

To retrospectively evaluate the results of the Food Program of the Autism Day Hospital during the year 2022.

**Methods:**

A retrospective analysis of the cases of patients admitted to the Food Program of the Autism Day Hospital during the year 2022 is carried out. Results of the sensory pattern and presence of genetic alterations of each one of the patients are compared. And the results of the intervention are evaluated by quantifying the new foods introduced into the diet at the end of the admission.

**Results:**

The sample is made up of a total of 5 children (4 boys and 1 girl) aged between 7 and 12 years. All of them meet diagnostic criteria for Autism Spectrum Disorder and present comorbidity with ARFID. Of the total sample, 1 of the patients presented in the genetic study a microdeletion S. in 15q13.3, duplication in 2q13 and duplication in 5p12-p11, with the genetic studies in the rest of the patients in the sample being normal. Regarding the results of the sensory pattern (Infant/Toddler Sensory profile test), all the patients presented differences in relation to other children of their age in the oral sensory pattern, this difference being definitive in 3 of the 5 patients in the sample. All the patients included in the program presented a satisfactory evolution, introducing at least 15-20 new foods into their usual diet, including different textures and consistencies.

**Conclusions:**

The therapeutic approach to ARFID in children with ASD carried out from a multidisciplinary perspective; sensory integration, behavioral approach and, if necessary, psychopharmacological, has shown, based on the results obtained from the food program of the ASD Day Hospital, a favorable evolution of the eating disorder. For this reason, we consider the detection of this typical comorbidity of ASD and its referral to specific therapeutic programs to be of special importance.

**Disclosure of Interest:**

None Declared

